# {(*E*)-2-Bromo-4-chloro-6-[3-(dimethyl­ammonio)propyl­imino­meth­yl]­phenol­ato}­dichloridozinc(II)

**DOI:** 10.1107/S1600536808016255

**Published:** 2008-06-07

**Authors:** Li-Juan Ye, Zhonglu You

**Affiliations:** a Department of Chemistry and Life Sciences, Xiangnan University, Chenzhou 423000, People’s Republic of China; b Department of Chemistry, Liaoning Teachers’ University, Dalian 116029, People’s Republic of China

## Abstract

The title compound, [ZnCl_2_(C_12_H_16_BrClN_2_O)], is a mononuclear zinc(II) complex. The Zn^II^ atom is four-coordinate in a tetra­hedral geometry, binding to the phenolate O and imine N atoms of the zwitterionic Schiff base ligand and to two Cl^−^ ions. In the crystal structure, mol­ecules are linked through inter­molecular N—H⋯Cl hydrogen bonds to form chains running along the *a* axis.

## Related literature

For related structures, see: Ali *et al.* (2008 ▶); Wang (2007 ▶); You (2005 ▶). For our recent investigations of metal complex systems, see: Ye & You (2007*a* ▶,*b* ▶,*c* ▶).
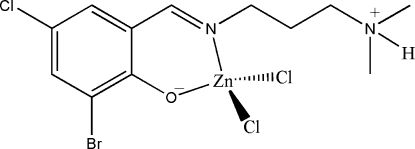


## Experimental

### 

#### Crystal data

[ZnCl_2_(C_12_H_16_BrClN_2_O)]
*M* *_r_* = 455.90
Monoclinic, 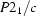

*a* = 7.522 (4) Å
*b* = 26.808 (15) Å
*c* = 8.354 (4) Å
β = 90.921 (9)°
*V* = 1684.3 (16) Å^3^
*Z* = 4
Mo *K*α radiation
μ = 4.30 mm^−1^
*T* = 298 (2) K
0.32 × 0.30 × 0.30 mm

#### Data collection

Bruker SMART CCD area-detector diffractometer
Absorption correction: multi-scan (*SADABS*; Sheldrick, 1996 ▶) *T*_min_ = 0.261, *T*_max_ = 0.275
9470 measured reflections
3633 independent reflections
2283 reflections with *I* > 2σ(*I*)
*R*_int_ = 0.037

#### Refinement

*R*[*F* ^2^ > 2σ(*F* ^2^)] = 0.048
*wR*(*F* ^2^) = 0.126
*S* = 1.04
3633 reflections
186 parameters
1 restraint
H atoms treated by a mixture of independent and constrained refinement
Δρ_max_ = 0.58 e Å^−3^
Δρ_min_ = −0.36 e Å^−3^

### 

Data collection: *SMART* (Bruker, 2002 ▶); cell refinement: *SAINT* (Bruker, 2002 ▶); data reduction: *SAINT*; program(s) used to solve structure: *SHELXTL* (Sheldrick, 2008 ▶); program(s) used to refine structure: *SHELXTL*; molecular graphics: *SHELXTL*; software used to prepare material for publication: *SHELXTL*.

## Supplementary Material

Crystal structure: contains datablocks global, I. DOI: 10.1107/S1600536808016255/sj2511sup1.cif

Structure factors: contains datablocks I. DOI: 10.1107/S1600536808016255/sj2511Isup2.hkl

Additional supplementary materials:  crystallographic information; 3D view; checkCIF report

## Figures and Tables

**Table:** 

Zn1—O1	1.931 (4)
Zn1—N1	1.999 (4)
Zn1—Cl2	2.2303 (19)
Zn1—Cl3	2.2489 (19)

**Table:** 

O1—Zn1—N1	95.48 (17)
O1—Zn1—Cl2	112.60 (13)
N1—Zn1—Cl2	112.04 (14)
O1—Zn1—Cl3	110.85 (13)
N1—Zn1—Cl3	114.71 (13)
Cl2—Zn1—Cl3	110.45 (8)

**Table 2:** Hydrogen-bond geometry (Å, °)

*D*—H⋯*A*	*D*—H	H⋯*A*	*D*⋯*A*	*D*—H⋯*A*
N2—H2⋯Cl3^i^	0.91 (5)	2.37 (3)	3.190 (5)	152 (5)

Symmetry code: (i) 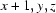.

**Fig. 1.**
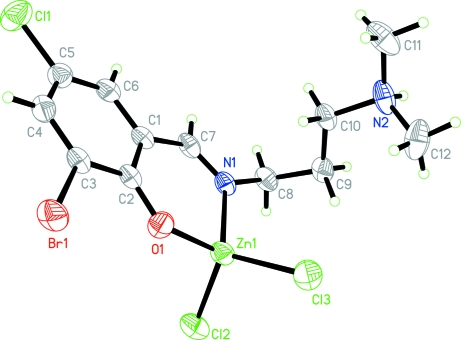
Molecular structure of (I) with displacement ellipsoids drawn at the 30% probability level.

**Fig. 2.**
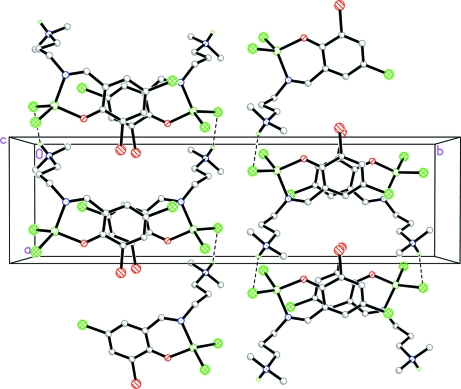
Molecular packing of (I). Intermolecular hydrogen bonds are shown as dashed lines.

## supplementary crystallographic information

### Comment

Recently, we have reported  thiocyanate coordinated zinc(II) (Ye & You, 2007*a*), and copper(II) complexes (Ye & You, 2007*b*), and a chlorido-bridged polynuclear copper(II) complex (Ye & You, 2007*c*). As an extension of the work on the crystal structures of such complexes, we report herein the crystal structure of the title compound, (I), Fig. 1.

Compound (I) is a mononuclear zinc(II) complex. The Zn^II^ atom is four-coordinate in a tetrahedral geometry, binding to the phenolate O and imine N atoms of the zwitterionic Schiff base ligand and two Cl^-^ ions. The coordinate bond values (Table 1) are comparable to those reported in other similar zinc(II) complexes (Wang, 2007; Ali *et al.*, 2008; You, 2005).

In the crystal structure, molecules are linked through intermolecular N–H···Cl hydrogen bonds, Table 2, to form chains running along the *a* axis (Fig. 2).

### Experimental

3-Bromo-5-chlorosalicylaldehyde (0.1 mmol, 23.5 mg), *N*,*N*-dimethylpropane-1,3-diamine (0.1 mmol, 10.2 mg), and zinc(II) chloride (0.1 mmol, 13.6 mg) were dissolved in a methanol solution (10 ml). The mixture was stirred at room temperature for 30 min to give a clear colorless solution. Crystals of the compound were formed by slow evaporation of the solvent over a week at room temperature.

### Refinement

Atom H2 on the amine N2 atom was located from a difference Fourier map and refined isotropically, with the N–H distance restrained to 0.90 (1) Å, and with *U*_iso_(H) fixed at 0.08 Å^2^. The remaining H atoms were placed in geometrically idealized positions and constrained to ride on their parent atoms with C–H distances in the range 0.93–0.97 Å, and with *U*_iso_(H) = 1.2 or 1.5*U*_eq_(C).

### Crystal data

**Table:**

[ZnCl_2_(C_12_H_16_BrClN_2_O)]	*F*_000_ = 904
*M**_r_* = 455.90	*D*_x_ = 1.798 Mg m^−^^3^
Monoclinic, *P*2_1_/*c*	Mo *K*α radiation λ = 0.71073 Å
Hall symbol: -P 2ybc	Cell parameters from 1372 reflections
*a* = 7.522 (4) Å	θ = 2.3–25.3º
*b* = 26.808 (15) Å	µ = 4.30 mm^−^^1^
*c* = 8.354 (4) Å	*T* = 298 (2) K
β = 90.921 (9)º	Block, colorless
*V* = 1684.3 (16) Å^3^	0.32 × 0.30 × 0.30 mm
*Z* = 4	

### Data collection

**Table:**

Bruker SMART CCD area-detector diffractometer	3633 independent reflections
Radiation source: fine-focus sealed tube	2283 reflections with *I* > 2σ(*I*)
Monochromator: graphite	*R*_int_ = 0.037
*T* = 298(2) K	θ_max_ = 27.0º
ω scans	θ_min_ = 1.5º
Absorption correction: multi-scan(SADABS; Sheldrick, 1996)	*h* = −9→9
*T*_min_ = 0.261, *T*_max_ = 0.275	*k* = −32→34
9470 measured reflections	*l* = −10→7

### Refinement

**Table:**

Refinement on *F*^2^	Secondary atom site location: difference Fourier map
Least-squares matrix: full	Hydrogen site location: inferred from neighbouring sites
*R*[*F*^2^ > 2σ(*F*^2^)] = 0.048	H atoms treated by a mixture of independent and constrained refinement
*wR*(*F*^2^) = 0.126	*w* = 1/[σ^2^(*F*_o_^2^) + (0.0506*P*)^2^ + 2.1641*P*] where *P* = (*F*_o_^2^ + 2*F*_c_^2^)/3
*S* = 1.04	(Δ/σ)_max_ = 0.001
3633 reflections	Δρ_max_ = 0.58 e Å^−^^3^
186 parameters	Δρ_min_ = −0.36 e Å^−^^3^
1 restraint	Extinction correction: none
Primary atom site location: structure-invariant direct methods	

### Special details

**Table:**

Geometry. All e.s.d.'s (except the e.s.d. in the dihedral angle between two l.s. planes) are estimated using the full covariance matrix. The cell e.s.d.'s are taken into account individually in the estimation of e.s.d.'s in distances, angles and torsion angles; correlations between e.s.d.'s in cell parameters are only used when they are defined by crystal symmetry. An approximate (isotropic) treatment of cell e.s.d.'s is used for estimating e.s.d.'s involving l.s. planes.
Refinement. Refinement of *F*^2^ against ALL reflections. The weighted *R*-factor *wR* and goodness of fit *S* are based on *F*^2^, conventional *R*-factors *R* are based on *F*, with *F* set to zero for negative *F*^2^. The threshold expression of *F*^2^ > σ(*F*^2^) is used only for calculating *R*-factors(gt) *etc*. and is not relevant to the choice of reflections for refinement. *R*-factors based on *F*^2^ are statistically about twice as large as those based on *F*, and *R*- factors based on ALL data will be even larger.

### Fractional atomic coordinates and isotropic or equivalent isotropic displacement parameters (Å^2^)

**Table:**

	*x*	*y*	*z*	*U*_iso_*/*U*_eq_	
Zn1	0.25129 (8)	0.59843 (2)	0.53367 (8)	0.0531 (2)	
Br1	−0.09048 (7)	0.73965 (3)	0.73104 (8)	0.0691 (2)	
Cl1	0.4472 (2)	0.86120 (6)	0.5360 (2)	0.0868 (6)	
Cl2	0.1025 (2)	0.56893 (7)	0.32087 (19)	0.0748 (5)	
Cl3	0.2609 (2)	0.54111 (6)	0.7302 (2)	0.0718 (4)	
O1	0.1550 (5)	0.66062 (14)	0.6102 (5)	0.0592 (10)	
N1	0.4876 (5)	0.62653 (18)	0.4750 (5)	0.0485 (10)	
N2	0.8671 (7)	0.5717 (2)	0.8271 (6)	0.0708 (15)	
C1	0.3975 (6)	0.7126 (2)	0.5273 (6)	0.0465 (12)	
C2	0.2268 (6)	0.7039 (2)	0.5936 (6)	0.0464 (12)	
C3	0.1364 (6)	0.7472 (2)	0.6440 (6)	0.0499 (13)	
C4	0.2038 (7)	0.7947 (2)	0.6301 (6)	0.0554 (14)	
H4	0.1408	0.8221	0.6675	0.066*	
C5	0.3661 (7)	0.8009 (2)	0.5599 (7)	0.0561 (14)	
C6	0.4609 (7)	0.7608 (2)	0.5106 (7)	0.0545 (14)	
H6	0.5712	0.7658	0.4645	0.065*	
C7	0.5140 (6)	0.6730 (2)	0.4739 (6)	0.0501 (13)	
H7	0.6226	0.6832	0.4335	0.060*	
C8	0.6332 (7)	0.5932 (2)	0.4223 (7)	0.0566 (14)	
H8A	0.7281	0.6131	0.3776	0.068*	
H8B	0.5886	0.5709	0.3394	0.068*	
C9	0.7047 (7)	0.5631 (2)	0.5607 (7)	0.0562 (14)	
H9A	0.8007	0.5420	0.5245	0.067*	
H9B	0.6115	0.5418	0.6012	0.067*	
C10	0.7721 (7)	0.5967 (2)	0.6924 (6)	0.0557 (14)	
H10A	0.8515	0.6211	0.6462	0.067*	
H10B	0.6717	0.6147	0.7353	0.067*	
C11	0.9452 (10)	0.6088 (3)	0.9358 (8)	0.094 (2)	
H11A	1.0131	0.6323	0.8755	0.140*	
H11B	1.0216	0.5924	1.0125	0.140*	
H11C	0.8522	0.6259	0.9906	0.140*	
C12	0.7535 (10)	0.5353 (3)	0.9111 (9)	0.092 (2)	
H12A	0.8145	0.5236	1.0058	0.137*	
H12B	0.7283	0.5075	0.8417	0.137*	
H12C	0.6442	0.5510	0.9404	0.137*	
H2	0.957 (6)	0.5538 (19)	0.785 (7)	0.080*	

### Atomic displacement parameters (Å^2^)

**Table:**

	*U*^11^	*U*^22^	*U*^33^	*U*^12^	*U*^13^	*U*^23^
Zn1	0.0387 (3)	0.0640 (4)	0.0565 (4)	0.0016 (3)	0.0037 (3)	0.0026 (3)
Br1	0.0423 (3)	0.0841 (5)	0.0814 (5)	0.0067 (3)	0.0171 (3)	−0.0044 (4)
Cl1	0.0821 (12)	0.0638 (10)	0.1152 (15)	−0.0146 (9)	0.0261 (10)	0.0035 (10)
Cl2	0.0598 (9)	0.1019 (13)	0.0625 (10)	−0.0042 (9)	−0.0082 (7)	−0.0015 (9)
Cl3	0.0628 (9)	0.0818 (11)	0.0713 (10)	0.0011 (8)	0.0118 (7)	0.0115 (9)
O1	0.045 (2)	0.056 (2)	0.077 (3)	0.0007 (18)	0.0174 (18)	0.005 (2)
N1	0.033 (2)	0.062 (3)	0.051 (3)	0.009 (2)	0.0019 (18)	0.003 (2)
N2	0.057 (3)	0.095 (4)	0.061 (3)	0.035 (3)	0.007 (2)	0.016 (3)
C1	0.033 (2)	0.064 (3)	0.042 (3)	0.002 (2)	−0.002 (2)	0.001 (3)
C2	0.034 (2)	0.062 (4)	0.043 (3)	0.006 (2)	−0.002 (2)	0.005 (3)
C3	0.036 (3)	0.067 (4)	0.046 (3)	0.006 (2)	0.000 (2)	0.006 (3)
C4	0.056 (3)	0.058 (4)	0.052 (3)	0.009 (3)	−0.004 (3)	−0.001 (3)
C5	0.050 (3)	0.060 (4)	0.058 (4)	−0.002 (3)	0.001 (3)	0.004 (3)
C6	0.039 (3)	0.070 (4)	0.055 (3)	−0.003 (3)	−0.002 (2)	0.004 (3)
C7	0.032 (2)	0.077 (4)	0.041 (3)	0.008 (3)	0.006 (2)	0.008 (3)
C8	0.040 (3)	0.072 (4)	0.058 (4)	0.014 (3)	0.006 (2)	−0.004 (3)
C9	0.040 (3)	0.061 (3)	0.068 (4)	0.014 (3)	0.011 (3)	0.000 (3)
C10	0.041 (3)	0.072 (4)	0.054 (3)	0.011 (3)	0.002 (2)	0.012 (3)
C11	0.081 (5)	0.155 (7)	0.044 (4)	0.008 (5)	−0.010 (3)	−0.003 (4)
C12	0.109 (6)	0.084 (5)	0.083 (5)	0.028 (4)	0.034 (4)	0.031 (4)

### Geometric parameters (Å, °)

**Table:**

Zn1—O1	1.931 (4)	C4—H4	0.9300
Zn1—N1	1.999 (4)	C5—C6	1.359 (8)
Zn1—Cl2	2.2303 (19)	C6—H6	0.9300
Zn1—Cl3	2.2489 (19)	C7—H7	0.9300
Br1—C3	1.877 (5)	C8—C9	1.502 (8)
Cl1—C5	1.740 (6)	C8—H8A	0.9700
O1—C2	1.288 (6)	C8—H8B	0.9700
N1—C7	1.261 (7)	C9—C10	1.504 (8)
N1—C8	1.485 (6)	C9—H9A	0.9700
N2—C11	1.464 (9)	C9—H9B	0.9700
N2—C12	1.482 (8)	C10—H10A	0.9700
N2—C10	1.483 (7)	C10—H10B	0.9700
N2—H2	0.91 (5)	C11—H11A	0.9600
C1—C6	1.385 (8)	C11—H11B	0.9600
C1—C2	1.425 (7)	C11—H11C	0.9600
C1—C7	1.451 (7)	C12—H12A	0.9600
C2—C3	1.413 (7)	C12—H12B	0.9600
C3—C4	1.375 (8)	C12—H12C	0.9600
C4—C5	1.372 (8)		
			
O1—Zn1—N1	95.48 (17)	N1—C7—C1	128.6 (5)
O1—Zn1—Cl2	112.60 (13)	N1—C7—H7	115.7
N1—Zn1—Cl2	112.04 (14)	C1—C7—H7	115.7
O1—Zn1—Cl3	110.85 (13)	N1—C8—C9	110.6 (4)
N1—Zn1—Cl3	114.71 (13)	N1—C8—H8A	109.5
Cl2—Zn1—Cl3	110.45 (8)	C9—C8—H8A	109.5
C2—O1—Zn1	125.6 (3)	N1—C8—H8B	109.5
C7—N1—C8	118.4 (4)	C9—C8—H8B	109.5
C7—N1—Zn1	121.0 (3)	H8A—C8—H8B	108.1
C8—N1—Zn1	120.6 (4)	C8—C9—C10	110.7 (5)
C11—N2—C12	112.5 (6)	C8—C9—H9A	109.5
C11—N2—C10	110.3 (5)	C10—C9—H9A	109.5
C12—N2—C10	112.5 (5)	C8—C9—H9B	109.5
C11—N2—H2	108 (4)	C10—C9—H9B	109.5
C12—N2—H2	106 (4)	H9A—C9—H9B	108.1
C10—N2—H2	107 (4)	N2—C10—C9	115.9 (5)
C6—C1—C2	120.3 (5)	N2—C10—H10A	108.3
C6—C1—C7	116.1 (5)	C9—C10—H10A	108.3
C2—C1—C7	123.6 (5)	N2—C10—H10B	108.3
O1—C2—C3	120.3 (4)	C9—C10—H10B	108.3
O1—C2—C1	124.7 (5)	H10A—C10—H10B	107.4
C3—C2—C1	115.0 (5)	N2—C11—H11A	109.5
C4—C3—C2	123.7 (5)	N2—C11—H11B	109.5
C4—C3—Br1	118.2 (4)	H11A—C11—H11B	109.5
C2—C3—Br1	118.1 (4)	N2—C11—H11C	109.5
C5—C4—C3	118.7 (5)	H11A—C11—H11C	109.5
C5—C4—H4	120.7	H11B—C11—H11C	109.5
C3—C4—H4	120.7	N2—C12—H12A	109.5
C6—C5—C4	120.5 (5)	N2—C12—H12B	109.5
C6—C5—Cl1	121.0 (4)	H12A—C12—H12B	109.5
C4—C5—Cl1	118.5 (5)	N2—C12—H12C	109.5
C5—C6—C1	121.7 (5)	H12A—C12—H12C	109.5
C5—C6—H6	119.2	H12B—C12—H12C	109.5
C1—C6—H6	119.2		

### Hydrogen-bond geometry (Å, °)

**Table:**

*D*—H···*A*	*D*—H	H···*A*	*D*···*A*	*D*—H···*A*
N2—H2···Cl3^i^	0.91 (5)	2.37 (3)	3.190 (5)	152 (5)

Symmetry codes: (i) *x*+1, *y*, *z*.
